# Identifying the Effects of Fish and Rhizosphere on the Structure of the Planktonic Bacterial Communities and Resistome in an Aquaponics Recirculation Loop

**DOI:** 10.1111/1758-2229.70128

**Published:** 2025-07-14

**Authors:** Frédérique Changey, Christophe Merlin, Camille Fourrier, Pascal Fontaine, Laurence Mathieu

**Affiliations:** ^1^ Université de Lorraine, CNRS, LCPME Nancy France; ^2^ University of Lorraine, INRAE, L2A Nancy France; ^3^ EPHE, PSL, UMR CNRS 7564, LCPME Nancy France

**Keywords:** aquaponic compartments, aquaponics, bacterial communities, bacterial drivers, resistome

## Abstract

Since they are saving resources, recirculating aquaculture systems (RAS) are perceived as a viable production strategy. Among them, aquaponics combines aquaculture and hydroponic vegetable production. This system entails a closed‐loop water system and continuity between two main “generators of bacterial biodiversity” the plant and the fish, respectively associated with the rhizosphere and the gut microbiome. These ecosystems are powerful bioreactors capable of shaping microbial community structures by mimicking macroscale environmental interactions through selection, competition, and genetic invasion. This work aimed to clarify the respective roles of these bioreactors on planktonic bacteria and antibiotic‐resistant bacteria (ARB) composition and segregation within a commercial aquaponic system composed of four aquaponics units differing by the fish reared. These units were assessed using a high‐throughput qPCR (HT‐qPCR) analysis of 384 antibiotic resistant genes (ARGs) and mobile genetic elements (MGEs) as well as 16S rRNA metabarcoding. The results highlight that the most important driver of bacterial communities and ARG distribution pattern in aquaponics environments appears to be the fish through their associated microbiota. Rhizosphere micro‐environment seems to act as a mitigation factor on the relative abundance of detectable ARGs.

## Graphical abstract

These results suggest that fish traits drive planktonic microbial communities. Rhizosphere seems to act as a mitigation factor on microbial communities and the relative abundance of detectable Antibiotic Resistance Genes.

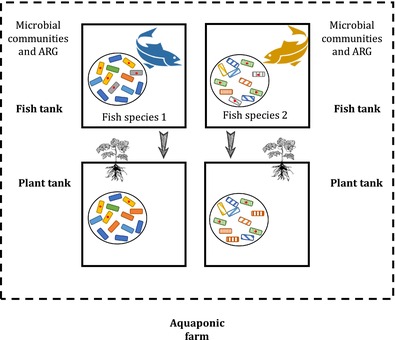

## 1 Introduction

Water scarcity and the pollution of resources, combined with an increase in global water consumption, have become significant challenges for humankind (Gosling and Arnell 2016; Ricart and Rico 2019). The search for a suitable eco‐friendly option for water reuse and water recirculation technology has thus been explored globally, especially in the agricultural and aquacultural sectors (Lavrnić et al. 2017). In this respect, aquaponics is an integrated circular system based on low water volume turnover that combines aquaculture and hydroponic plant production (McMurtry et al. 1997) while eliminating their respective disadvantages (e.g., the cost of fertilisers for hydroponics and aquaculture effluent elimination and treatment). Although chemically enriched water is the usual method of hydroponic plant cultivation, aquaponics allows essential nutrients to be received from fish waste by means of Recirculating Aquaculture System (RAS) (Rakocy 2012; Goddek et al. 2019). From a technical perspective, an aquaponic unit setup consists of a fish tank, followed by a biofilter unit to improve nitrification, and hydroponic culture units dedicated to soilless plant growth. To be effective, such recirculating systems must achieve an optimal balance of fish physiology, waste production, and nutrient consumption by plants, but this balance must also consider the microbial compartment. The success of the aquaponics system depends, therefore, in part on operationally controlling microbial activities. The microbial communities must therefore be treated as a keystone because they ensure high water quality by, for example, degrading waste and converting nitrogen into a non‐toxic form (Schmautz et al. 2017). Significant efforts have been devoted to the study of the fish and plant, which play a central role in the functioning of systems in which they are embedded, and numerous publications have also addressed the microbial communities' composition of RAS units, with a predominant focus on the biofiltration process (Schreier et al. 2010; Jiang et al. 2019). However, there remains a gap of knowledge concerning the complexity of microbial interactions (Kushwaha et al. 2025) and the dependency of the entire ecosystem on certain critical and low abundant microbial species (Somerville et al. 2014; Kasozi et al. 2021).

The aquaponic loop setting is considered as a series of functional modules (e.g., fish tanks and filters), each capable of independently influencing the overall planktonic microbial communities. Previous study reported that each compartment functioned as an independent microbial community, despite being in constant communication with other compartments (Ruiz et al. 2023). Aquaponics water microbial communities are the result of inoculation differing from the compartments (e.g., fish, plants, and water) and selective effect. Among the inoculation sources, fish have been identified as a microbial gateway to the aquaponic system (Yukgehnaish et al. 2020). Bacterial transfer between fish microbiota and the surrounding environment has been previously reported (Chen et al. 2022). However, planktonic and sessile communities in aquaponic systems were found to be different from bacterial communities in the gut of farmed fish (Ruiz et al. 2023). Another study reported that in aquaponic systems, 75% of the genera are likely to be slow‐growing and predominant, whereas the few fast‐growing opportunists are mainly associated with the gut of farmed species (Moschos et al. 2022). Consequently, the role of fish species in influencing bacterial exchange with the surrounding water remains to be fully elucidated. In parallel, plants are also known to act as significant selective drivers of bacterial communities through their rhizospheres and carbon exudations (Compant et al. 2021; Ulbrich et al. 2022; DeAngelis et al. 2009). It has been demonstrated that a similar phenomenon occurs in the context of aquaponics. Other authors have shown that the root‐driven effect of plants is capable of shaping the bacterial communities in hydroponics and aquaponics (Lobanov et al. 2022). However, the extent to which these phenomena occur in soil‐less aquaponic systems remains to be extensively studied.

As a closed circuit, the aquaponic loop facilitates the efficient diffusion of microorganisms between compartments (fish, biofilters and plant) with specific hazards in the case of pathogen or antibiotic‐resistant bacteria (ARB). Indeed, in aquaculture systems, potential pathogens belonging to *Acinetobacter*, *Arcobacter*, and *Clostridium* have been identified (Xiong et al. 2015). Recent research has also reported that pathogens (*Aeromonas* or *Flavobacterium* genus) could be responsible for fish diseases (Rieder et al. 2023). In some ways, the dissemination of antibiotic resistance could be represented as an amplification loop that alternates between selection processes (antibiotic therapies) and contagion processes (the release of resistant bacteria into the surrounding environment). Despite their poor persistence outside of human and animal bodies, released ARB may spread antibiotic resistance genes (ARGs) into environmentally adapted microbes by horizontal gene transfer (Partridge et al. 2018). Accordingly, environmental bacterial communities have the potential to act as reservoirs of ARGs which could then be transferred to human and animal microbiota or even potential pathogens. Recent work, performed on aquaponics, has identified high levels of ARG despite the absence of antibiotic treatment (Kampouris et al. 2022).

All environments do not exhibit the same susceptibility to ARB and ARG contagion, as invading bacteria are confronted with residing autochthonous bacterial communities (Gionchetta et al. 2023; Leão et al. 2023). Aquaponics, in particular, poses a multifaceted challenge due to its involvement of diverse microbiota from fish, plants, and biofilters. Each of these compartments can fulfil distinct functions as an ARB carrier, an ARB growth promoter, or a barrier to ARB contagion. Little attention has been granted to obtaining a comprehensive overview of the potential invasion and behaviour, or simply, the distribution of ARB in RAS settings.

In this context, the primary objective was to ascertain the composition of bacterial communities within the aquaponic system by conducting a comprehensive sampling of the three distinct compartments: the aquarium, the biofilter, and the hydroponic section. This approach enabled the investigation of the impact of fish (monoculture vs. polyculture) and plants (rhizosphere) on bacterial community structures. The second objective was to study the fate of ARB/ARG in the system in order to identify the impact of these diversity generators (i.e., fish and the rhizosphere) on the system's resistome. This investigation was performed on an experimental aquaponic farm with four independent and similarly operated aquaponic units that differed only by fish species. (Carp, Goldfish in monoculture and Carp and goldfish in polyculture). The circulating planktonic communities were studied using a metabarcoding approach on the 16S rRNA gene, whereas the compartmentalization of the ARGs' resistome on the aquaponics farm was assessed with SmartChip high‐throughput qPCR.

## 2 Experimental Procedures

### 2.1 Experimental Area

**FIGURE 1:**
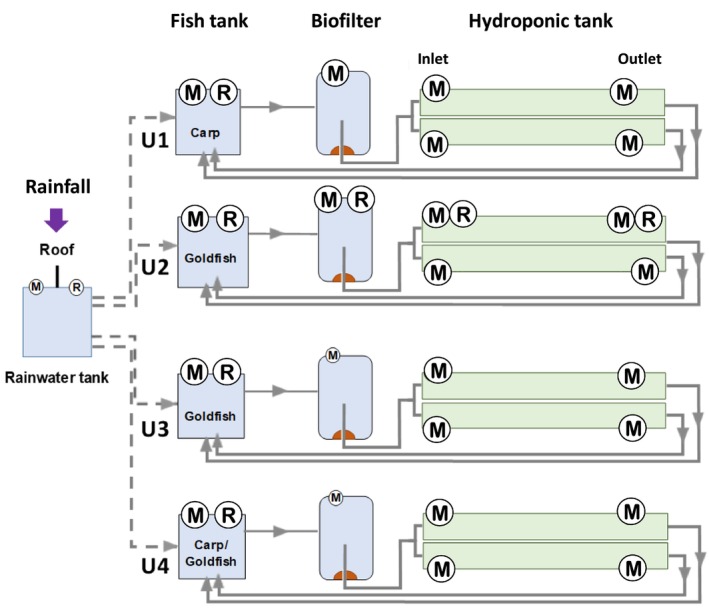
Scheme of the four aquaponic units with the different key compartments including the rainwater tank supply system. Circled “M” symbolise sampling points for metabarcoding analysis (fish tank, biofilter system and at the inlet and outlet of the two hydroponic sections); Circled “R” symbolise sampling points for HT‐qPCR Resistomap assays; Fish species reared in each of the 4 aquaponic units (U1, U2, U3, U4) are indicated.

The commercial aquaponic system farm studied is located in Chaumousey (Vosges, France; 48.16 N 6.34 W; Siret: 43482106200016). The 1550 m^2^ farm has been in operation since 01/01/2021. The system is operated using rainwater collected from the rooftop of the greenhouse hosting the aquaponic system and stored in a rainwater tank before being used to compensate for water loss within the systems (i.e., evaporation, leakage). Water from the fish tank was pumped (flow rate = 12 L min^−1^) and circulated continuously in a closed‐loop manner from the fish tanks to the hydroponic compartments and returned to the fish tank (Figure 1). This RAS system consists of fourindependent and similarly operated units of 50 m^3^ named U1, U2, U3, and U4, respectively. One unit consisted of a fish‐rearing tank, a biofilter filled with Kaldnes biofilm carriers, and 2 parallel hydroponic tanks for plant cultures. Three of the four units operated in fish monoculture mode, with Carp ( *Cyprinus carpio* ) for U1 and Goldfish ( *Carassius auratus* ) for U2 and U3. The U4 unit was operated in polyculture mode with Goldfish ( *Carassius auratus* ) and Koi Carp ( *Cyprinus carpio carpio* ). These fish species were selected by the farmer for their interest in the local market, their temperature tolerance (water temperature between 4°C and 26°C), and their omnivorous feeding behaviour. All fish tanks were filled with a fish density of 8 ± 1.5 kg m^−3^ and were aerated with a pump to reach a dissolved oxygen (DO) concentration above 6.6 mg/L. Fish were fed *ad libitum* with a commercial food (Aqua Bio, Belgium) composed of 70% protein, 7% crude fat, 5.7% ash, and 3% fibre to cover all nutrients required for the fish. In the hydroponics tanks, the following vegetable species were distributed randomly: Lettuce ( *Lactuca sativa* ), Squash ( *Cucurbita pepo* ), Beans ( *Vicia faba* ) and Chard ( *Beta vulgaris* ).

### 2.2 Samples Collection

Sampling was performed during the harvest season in a rotational planting system of market gardening. Water samples were collected in June 2021 in the upper 30 cm of the water column in the rainwater tank and in each of the four units at four points along the aquaponic continuum (Figure 1). For biomass quantification by flow cytometry, 50 mL of water was collected in sterile tubes (Falcon, Corning). For the metabarcoding community analyses, 500 mL of water was collected in disposable sterile PE bottles (Corning Life Science) in triplicate for each sampling point along the unit continuum: fish tank (*n* = 12), biofilter (*n* = 12), 2 hydroponic tank inlets (*n* = 24) and 2 hydroponic tank outlets (*n* = 24). Regarding the ARG distribution analysis by HT‐qPCR, only the four fish tanks of U1, U2, U3, and U4 and along the continuum of U2 (fish tank, biofilter and inlet and outlet of hydroponic tanks) were sampled, as well as the rainwater tank (Figure 1). All these water samples were then stored at −20°C until DNA extraction.

### 2.3 Quantification of Bacterial Biomass by Flow Cytometry

The total number of water bacteria was determined using flow cytometry (FCM) after bacterial staining with SYBR Green I Nucleic Acid Gel Stain (S7563, Invitrogen, France). From the water samples collected as mentioned above, 1 mL of water‐or its decimal dilution‐was labelled with the fluorochrome SYBR Green I (ex/em: 497 nm/520 nm; final concentration 1×). After 15 min of staining in the dark, FCM analysis was performed using an Accuri C6 Plus flow cytometer (BD Biosciences, USA) equipped with a blue laser at 488 nm. Ultrapure water was used as sheath fluid. SYBR‐I‐stained bacteria were detected using the FL1 detector (533 nm ± 30). Events were triggered on the forward scatter (FSC) parameter with a threshold of 2500 and on the FL1 detector with a threshold of 700. Flow cytometer data were acquired over 2 min at a flow rate of 35 μL min^−1^. FCM data were analysed using BD Accuri C6 software (BD Biosciences) and batch processed. Electronic gating was used to separate bacterial cells from background, to visualise cell distributions in FSC, SSC, FL1, and to calculate geometrical means of the total bacteria count.

### 2.4 DNA Extraction and Amplicon Sequencing

After thawing (overnight at 20°C–22°C), 100 mL of each water sample was filtered through a 0.2 μm membrane filter (Whatman Nuclepore Track‐Etched Membranes, Merck, Germany) using a vacuum filtration device. Community DNA extraction was performed using the DNeasy PowerWater kit (Qiagen, Hilden, Germany) according to the manufacturer's recommendations. DNA concentration was measured by the QuBit4 Fluorometer (Invitrogen, Waltham, USA) method. In addition, the purity of the DNA extractions was assessed by measuring both 260/280 nm and 260/230 nm ratios with a NanoDrop spectrophotometer (Thermo Scientific, Waltham, USA). Extracted DNA was stored at −20°C until use. Sequencing was performed of the V3‐V4 hypervariable regions of the bacterial 16S rRNA at the Illumina MiSeq platform from the Institute of Clinical Molecular Biology (IKMB, Kiel, Germany) using 357F [5′CCTACGGGAGGCAGCAG′3] and 806R [5′GACTACHVGGGTWTCTAAT′3] primers (Kozich et al. 2013).

### 2.5 Bioinformatic Analysis

Data were subsequently imported into the FROGS pipeline (Find Rapidly OTU with Galaxy Solution) implemented on a Galaxy instance (v.2.3.0) (http://sigenae‐workbench.toulouse.inra.fr/galaxy/) (Escudié et al. 2018). After a quality control, 16S paired‐end sequences were merged into contigs with VSEARCH (Rognes et al. 2016). Sequences were dereplicated before being clustered using the SWARM algorithm (v.2.1.5) (Mahé et al. 2022) with the first denoising step using an aggregation distance equal to 1 while the second step used a distance of 3. Chimeras were removed using VSEARCH (Rognes et al. 2016). Filters were applied to remove clusters that are not present in at least 3 samples or with an abundance below a 0.005% threshold (Rognes et al. 2016). The taxonomic assignment of each ASV was performed using the BLAST tools against (Camacho et al. 2009) the database SILVA 132 16S (Pruesse et al. 2007). The dataset was rarefied by randomly selecting subsets of sequences based on the sample with the fewest read counts. Phyloseq (1.26.1) R package was used to identify community composition analysis, to normalise and to generate α‐diversity indexes (richness and Shannon index) after a rarefaction curve using the transform counts method (Figure S3) (McMurdie and Holmes 2013).

### 2.6 High‐Throughput qPCR

Remaining DNA extraction samples were first diluted to 10 ng/μL. DNA extraction samples were sent to Resistomap Oy (Helsinki, Finland) for High‐Throughput qPCR analysis using the SmartChip Real‐Time PCR system (Takara Bio, Mountain View, CA, USA) targeting 383 ARG/MGE and 16S rRNA using a 384‐well template of primers. Twenty‐eight threshold cycles (CT) were used as cut‐off as previously suggested by previous analysis (Stedtfeld et al. 2018). DNA samples were analysed in 3 replicates qPCR reactions, and replicates were removed when they were only present in one technical replicate in order to exclude false positives. The 16S rRNA gene cycle threshold (Ct) was used to normalise the abundance of the other 383 quantified ARG/MGE genes using ΔCT method (where ΔCT = Ct of the targeted ARG—Ct of the 16S rRNA gene).

### 2.7 Statistical Analysis

All statistical analyses were carried out using R software version 3.3.1 (R Development Core Team, www.R‐project.org). We first compared α‐diversity indexes to summarise the structure of bacterial communities with respect to its richness number of Amplicon Sequence Variant (ASV) and Shannon index (to estimate the diversity of species within a community). After normality and homoscedasticity verifications (Shapiro (> 0.05) and Bartlett test (< 0.05) respectively), ANOVA (significance was declared at *p* < 0.05) was performed on richness and Shannon index, and Tukey HSD post hoc test was used for pairwise comparisons using “agricolae” package. The β‐diversity, to assess the degree of community differentiation, was estimated by using the relative abundance matrices and multivariate analyses. The dissimilarities (Bray Curtis distances) among treatments were assessed using Principal Coordinate Analysis (PCoA) followed by Between‐Class Analysis (BCA) using the “ade4TkGUI” package. PERMANOVA was performed using the “adonis” function (vegan 2.2.1 R package) to test the link between bacterial community composition obtained with the different experimental variables (9999 permutations). Plots were drawn using the R packages ggplot2 (3.1.1) and phyloseq (1.26.1) (McMurdie and Holmes 2013). Heatmaps were plotted with R package ampvis2 (2.5.1) (Andersen et al. 2018).

## 3 Results

### 3.1 Structure of the Circulating Bacterial Communities Throughout the Aquaponic Setting

The dynamics of bacterial communities is relevant for aquaponics because a well‐established and balanced bacterial community provides essential services both for fish and plant metabolism. Aquaponics is a recirculating system, with several interconnected compartments, that is fish and plant tanks, sustaining bacterial growth and therefore likely to influence the structure of the water‐circulating communities. This study aimed to map the bacterial biodiversity dynamics along the aquaponic continuum and identify the contribution of the fish and plant compartments to shaping the circulating bacterial community's and antibiotic‐associated resistome.

### 3.2 Bacterial Abundance Assessed by Flow Cytometry Along the Aquaponics Continuum

Preliminary, flow cytometry analyses showed that the total bacterial concentration was comprised between 4 × 10^5^ and 1 × 10^6^ bacterial cells/mL (Figure S1). There is no tendency nor significant variation in bacterial biomass between sampling points within the same aquaponic continuum. This suggests that the bacterial loads in the water of the different aquaponic units were similarly balanced and do not appear to be influenced by the fish and plants compartments. This implies that if any change in community structure is observed, it does not result from the singular increase or loss of bacterial biomass.

### 3.3 Bacterial Communities Change Along the Continuum and Between Aquaponics Units

**FIGURE 2:**
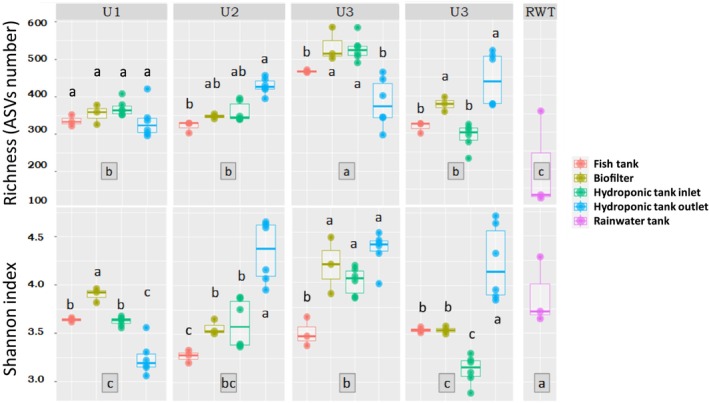
Microbial alpha‐diversity indexes across the different compartments of the four aquaponic units. The alpha‐diversity is presented as a measure of species richness (number of ASVs) and the Shannon Index to describe biodiversity. Different letters above the box‐plots indicate significant differences (*p* < 0.05) among compartments. The framed letters under each aquaponics unit indicate the results of the post hoc test on ANOVA carried out between each aquaponics unit (all compartments combined).

**FIGURE 3:**
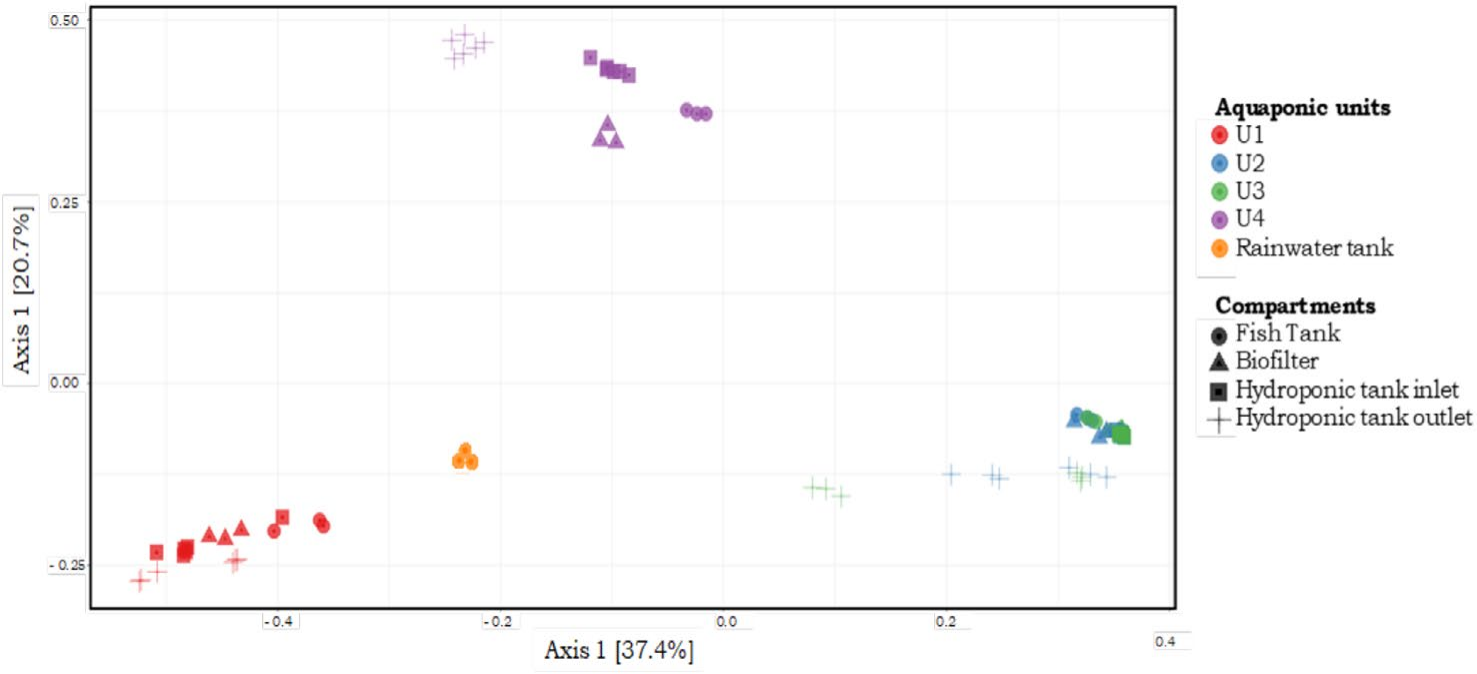
Principal coordinate analysis (PCoA) of distances between microbial communities based on Bray–Curtis distance among the 4 aquaponic units (colours) and the compartments (shapes).

**FIGURE 4:**
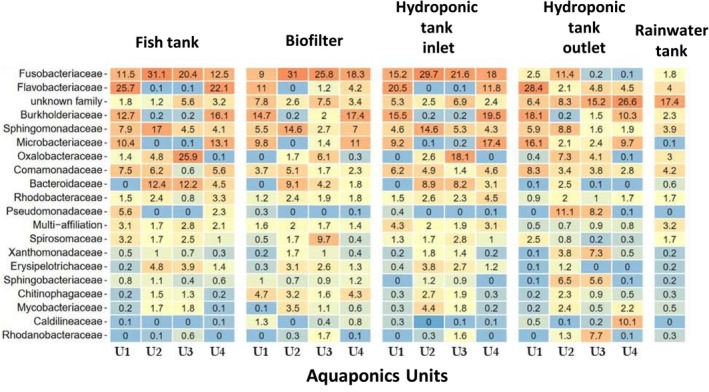
Composition and relative abundances of bacterial families among sampling points and aquaponic units. Colours are scaled from highest (red) to lowest (blue) values within columns.

The structure of the circulating bacterial communities was defined by metabarcoding analysis of community DNAs extracted from samples collected as shown in Figure 1. A total of 3,669,315 high quality sequences were obtained after a demultiplexing step (with an average of 25,145 sequences per sample). The sequences were grouped into 400 ASVs (97% nucleotide sequence identity threshold) and the flattening rarefaction curves (Figure S3) indicated sufficient sequencing depth, thus enabling biodiversity analyses (Figure S3). The overall α‐diversity results (i.e., bacterial ASVs richness and biodiversity Shannon indices to consider both species richness and specific diversity) for each aquaponic unit and compartments are presented in Figure 2. In the rainwater tank, the species richness is significantly lower and biodiversity higher than those observed for the fish tank into which it flows from time to time (regardless of the aquaponics units). Taken globally, the evolution of community richness and biodiversity along the continuum (between the four compartments of each aquaponic units) varies from one unit to another (*p* < 0.001). Principal coordinate analyses (PCoA) were used to identify bacterial communities clustering patterns according to the aquaponics units and compartments (Figure 3). Based on Bray–Curtis distances, PCoA revealed that β‐diversity featured significant separation on the first principal component (37.4% contribution) and the second principal component (20.7% contribution) axes. There were significant differences in bacterial community structure between different units (colour on the figure). Permutational Multivariate Analysis of Variance (PERMANOVA) confirmed that the observed clustering patterns in PCoAs were statistically significant (*p* = 0.0001). Clustering patterns highlight that the rainwater tank occupies a place between the 4 groups of units. It is also worth noting that units 2 and 3, both hosting Goldfishes in the fish tank, tended to cluster together, and that unit 4, which hosted both Goldfishes and Carps, seemed to group in between unit 1 (hosting carps) and 2–3 (hosting Goldfishes) on axis 1. PERMANOVA results also revealed that the bacterial structure between compartments within units was slightly but significantly different (*p* < 0.05) mainly due to the aquaponics tanks outlet. ASV assignation analysis (Figures 4 and S4) indicated that Fusobacteriaceae, Flavobactericeae and occasionally Oxalobacteraceae dominate the fish tank. Burkholderiaceae relative abundance remains always high across all compartments of U1 and U4. Fusobacteriaceae (and Fusobacterales at the order level; Figure S4) remains high in the biofilter and HT inlet, but its relative abundance decreases in the HT outlet of all units. The Pseudomonadales (Figure S4) are relatively abundant only in hydroponics tank outlet. Interestingly, in U2 and U3, in contrast to U1 and U4, the relative abundance of Flavobacteriaceae, Burkholderiaceae, and Microbacteriaceae is minimal with only slight increases in the HT outlet. Conversely, Bacteriodaceae has a relatively high abundance (> 8%) only in U2 and U3 (but not in the outlet).

Fusobacteriaceae remained elevated in the biofilter and the hydroponic inlet but drastically decreased in the hydroponic outlet. Besides, in U2 and U3, Pseudomonaceae, Xanthomonaceae, and Sphingobacteriaceae families are enhanced within the plant tanks. The U2 and U3 exhibit the same pattern for Pseudomonaceae that seem to be over‐selected at the end of the hydroponic tank. The rainwater tank, for its part, does not exhibit the same bacterial distribution as the rest of the entire aquaponic continuum, which was expected as this compartment has no real continuity with the other, only contributing for occasional water refill. The Venn diagram (performed with metabarcoding data) and presented in the (Figure S2) highlights that only 43% of ASVs are shared among aquaponic units (core microbiota) associated with this agricultural practice.

### 3.4 Structure and Spatial Evolution of the ARGs Resistome Along the Aquaponics Continuum

**FIGURE 5:**
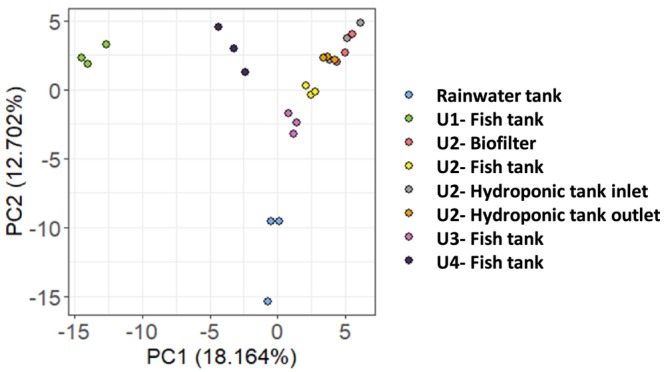
Principal Component Analysis (PCA) based on the relative abundances of Antibiotic Resistance Genes (ARGs) and Mobile Genetic Elements (MGEs) in water samples from all compartments of the U2 aquaponic unit (U2) and from all fish tanks across various aquaponic units.

**FIGURE 6:**
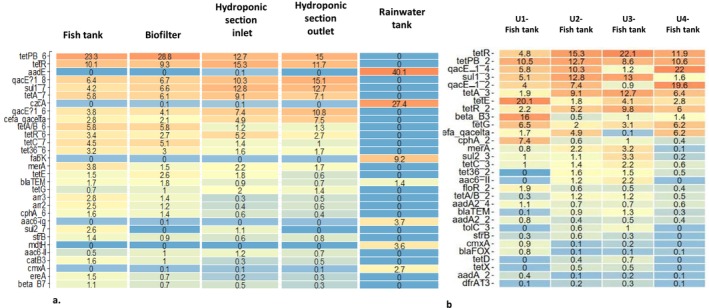
Resistome composition of the 30 highest ARG/MGEs and relative abundances in water (after normalisation by 16S rRNA gene) across the aquaponic unit 2 (a) and between the fish tanks of the 4 aquaponic units (b). Colours are scaled from highest (red) to lowest (blue) values within columns.

Based on the HT‐qPCR assay, 383 ARGs/MGEs multivariate analysis (PCA) were performed to compare the different ARGs/MGEs abundance profiles in the water planktonic bacterial communities within the compartments of unit U2 and across the fish tanks of the 4 aquaponic units (Figure 5). On the PCA explaining 30.8% of the variability, the rainwater tank resistome clustered apart. PCA also showed (i) that there are slight differences in the resistome along the U2 continuum, that is from fish tank to hydroponic tank outlet, (ii) that the resistome of the planktonic bacterial communities of the U2 fish tank is closer to that of the U3 fish tank. To go further, a heatmap was realised to represent the ARG with the 30‐highest relative abundance in Figure 6. This analysis was carried out twice independently: firstly, to assess the variation in composition and relative abundance of the main ARGs in unit 2 (Figure 6a) and secondly to assess the difference in these same parameters between the fish tanks of the 4 aquaponic units (Figure 6b). Among them, determinants for sulphonamides, cyclines, and aminoglycoside resistances appeared to be the dominant ARG class. The figure shows that the compartment with the greatest richness of ARGs (i.e., with a quantification cycle less than 28) is the fish tank. The composition and relative abundance of ARGs seem to change progressively along the aquatic continuum (from the fish tank to the hydroponic section tank) with a decrease in ARG/MGEs richness.

## 4 Discussion

The first aim of this study was to map changes in the bacterial communities' structure along the continuum of a commercial aquaponic farm (fish tank, biofilter, hydroponic section) and between four operational units (with different fish species), and to identify the driving contribution of the fish species and plant rhizosphere on circulating bacterial community and its associated resistome. Bacterial abundance was similarly balanced across the whole aquaponic systems (as indicated by flow cytometry results). Alpha and βeta‐diversity analysis showed that bacterial community's diversity and structure varied significantly between units and compartments.

Shannon diversity index analysis indicates a coherence between aquaponic units 2 and 3 (Figure 2). In terms of bacterial richness, these units host the highest number of amplicon sequence variants (ASVs). Overall, the variance in bacterial structure seems to be better explained by the effects of the aquaponic units rather than by the compartment's effect, highlighted by β‐diversity change. Indeed, multivariate analysis (Figure 3) has shown a convergence between units 2 and 3, which were distinct from the other units, indicating that they shared similar bacterial community structures. PERMANOVA confirmed this finding (*p* = 0.0011), which showed less divergence between units 2 and 3 than between other side‐by‐side comparisons. A Venn diagram showed that units 2 and 3 shared the highest proportion of ASVs (75%). This result confirms that the microbial communities in these two units are very similar.

Interestingly, unit 4, which houses goldfish and carp, appears to host a hybrid bacterial community. Fish could be important drivers, as they can excrete microorganisms via their faeces, gills, or epidermal mucus, which may then be transferred to the surrounding environment (Muziasari et al. 2017). Several ASVs are known to be shared among different fish species (Givens et al. 2015), with certain bacterial species specifically associated with individual fish species and their life histories. Bacterial composition analysis could help to support this hypothesis; the Fusobacteriaceae and Flavobacteriaceae families (which are poorly represented in the rainwater tank) dominate the bacterial communities in fish tank water compared to the hydroponic section outlet. Indeed, the Fusobacteriaceae family consists of gram‐negative rods found in the oral and intestinal mucosa, including the *Fusobacterium* genus, which is abundant in fish guts and faeces (Larsen et al. 2014; Schmautz et al. 2017). These bacteria are not described as being capable of long‐term persistence in aerobic environments (as they are only aerotolerant) their occurrence has been reported in biofilters of aquaponics systems (Itoi et al. 2007). The decrease in their relative abundance in the outlet of the hydroponic tank could be the result of their inability to withstand different environments. At the order level, bacteria belonging to the order Bacteroidales and Enterobacterales hosting numerous species of the intestinal microbiota (Figure S4) and seemed to be negatively selected as they move away from the fish tank. In 2020, a study established that half of the operational taxonomic units (OTUs) detected in surrounding water samples were also present in the intestinal microbiota of the *Hypophthalmichthys molitrix* (Zeng et al. 2020). This finding suggests that colonisation processes—albeit dynamic—are occurring in the system. The influence of fish on planktonic aquaponics bacterial communities could be indirect, stemming from metabolic activities such as ammonium excretion, or direct, through the emission of bacteria from fish microbiota. The composition of fish microbiota has been shown to depend on species and host selection effects, diet, environmental factors, habitat, and feeding behaviour (Muziasari et al. 2017; Chen et al. 2022). Numerous studies have reported that fish microbiota vary based on diet (carnivorous vs. herbivorous and natural vs. artificial) (Nyholm et al. 2022).

In the literature, studies have reported very strong effects of biofilters on the structure of bacterial communities, whereas in our study, this effect is not very clear‐cut. On the other hand, multivariate analysis and relative abundance studies show that communities change significantly between the inlet and outlet of the hydroponic tank, accompanied by an increase in the Shannon α‐diversity index. In soil, the rhizosphere, defined as the region around plant roots, is known to exert pressure on their associated communities through molecular interactions (e.g., exudates, antimicrobials, and quorum‐sensing signals) (Swamy et al. 2016; Fan et al. 2017). However, little is known about how these dynamics operate in soilless cultures with water recirculation. In aquaponics, hypothetically, two factors may limit the effects of the rhizosphere: (i) the diffusion of rhizosphere molecules occurs in large volumes of water, and (ii) contact with planktonic bacteria is transient due to water recirculation. This finding shows that plants could exert a selective influence on planktonic bacterial communities in hydroponics culture conditions. A study has highlighted the same trend on the rhizosphere lettuce compartment in shaping bacterial communities composition in aquaponics (Lobanov et al. 2022). This rhizosphere driving force also appears to extend to the associated resistome, which was reduced at the rhizosphere‐water interface, as indicated by a slight decrease in the relative abundance of detectable ARGs and mobile genetic elements (ARGs and MGEs) in the hydroponic outlet.

The contribution of exogenous bacteria, ARGs, and MGEs from the rainwater tank seems moderate. The tank‐stored rainwater harboured some ARGs/MGEs (mainly the inner membrane transporter (CzcA) and aminoglycoside *resistance genes* (*aadE*)) but with a distribution pattern that differs from the resistome of the 4 fish tanks bacterial communities. This could suggest that the bacteria carrying these ARGs/MGEs in the rainwater tank have been under‐selected in the aquaponics system. This finding can be attributed to approximately 10–20 L of water being added daily to the rainwater tank to compensate for leaks and evapotranspiration, even though it contained the same water that filled the tanks a year ago. However, this bacterial contribution is insufficient to induce a shift in the bacterial communities and resistome composition of a unit with over 10,000 L. The resistome distributions of fish tanks 2 and 3 appeared relatively similar, whereas unit 1, which hosts a different fish species (carp), exhibited a more singular ARG composition (Figure 6). Notably, aquaponic unit 1 shows distinct community structures and a singular resistome. The resistome composition displayed two interesting patterns. First, the relative abundance of ARGs decreased along the aquaponic continuum from the fish tank to the hydroponic section outlet in unit 2 (Figure 6). This observation leads to the second point highlighted in the data: the correlation between the resistome and the taxonomy of bacterial communities in aquaponics systems.

Our findings indicate that it seems to have a relationship between the distribution of ARGs and the composition of bacterial communities. Such relationships have been previously reported; for instance, ARGs have shown significant positive correlations with specific bacterial taxa in co‐occurrence network analyses (Zhang et al. 2020). Another study made similar observations, noting that Actinobacteria and Firmicutes were the most prolific spreaders of ARGs under their experimental conditions (Huerta et al. 2013). Other publications have explored correlations between ARGs and phylogeny, as recently synthesised by Liu et al. (2019) (Liu et al. 2019), and (Forsberg et al. 2014; Feng et al. 2018) who are able to rely on some specific bacterial taxa and specific ARGs (Forsberg et al. 2014; Feng et al. 2018). Our study supports the hypothesis that, in aquaponic systems, certain bacterial species are specifically associated with particular ARGs and that the structural variation of bacterial communities will imprint these changes on the resistome. Some ARGs have a chromosomal localization which is inheritable through vertical transmission. This makes sense in light of the fact that there is a relationship between bacterial communities and resistome diversity. But this relationship does not explain all the resistome variability in our experiment. Indeed, the other part could be attributable to horizontal gene transfer within the aquaponics system and deserves to be understood as for aquaculture (Preena et al. 2020).

## 5 Conclusion

In aquaculture and even more in aquaponics, the resistome and its potential risk for human health is not well documented. For instance, residual levels of antibiotics in fish and shellfish, transfer rates of ARB to fish and vegetables are not measured and depend on the farm and the practice. This study allowed us to identify that the hydroponic compartment (on which the plants grown) could allow a reduction in the richness of ARGs detectable in the water. The second striking fact is that the species of fish (or their life history) appears to be an important driver of bacterial communities and the resistome in aquaponics. Shifts in resistome composition could be the result of bacterial selection by the local environment, maybe associated with horizontal gene transfer.

## Author Contributions

**Frédérique Changey:** conceptualization, methodology, software, writing – original draft, data curation, formal analysis, investigation. **Christophe Merlin:** validation, writing – review and editing, methodology, conceptualization. **Camille Fourrier:** investigation, resources, writing – review and editing, data curation. **Pascal Fontaine:** supervision, project administration, funding acquisition, writing – review and editing. **Laurence Mathieu:** methodology, formal analysis, validation, investigation, writing – review and editing, conceptualization.

## Conflicts of Interest

All authors certify that they have no affiliations with or involvement in any organisation or entity with any financial or non‐financial interest in the subject matter or materials discussed in this manuscript. No approval from research ethics committees was required to accomplish this study as it did not involve any animal experimentation. The datasets generated during the current study are available from the corresponding author upon reasonable request.

## Supporting information


**Figure S1.** Total bacterial biomass (cells/mL) quantified by flow cytometry after Sybr Green staining within the circulating water of the different compartments of the 4 aquaponics units (*n* = 2–4 replicates).
**Figure S2.** Venn diagrams showing the unique and shared ASVs of the bacterial communities between different aquaponics units.
**Figure S3.** Rarefaction curve analysis showing the depth of 16S rRNA gene sequencing.
**Figure S4.** Microbiome composition and relative abundances of bacterial family among sampling point and aquaponic units. Colours are scaled from highest (red) to lowest (blue) values within columns.

## Data Availability

The data that support the findings has been deposited at the following URL: https://ent.univ‐lorraine.fr/f/u24l1s4/p/esup‐filemanager.u24l1n67/max/render.uP?pCm=view.
