# Supplementary material for: Identifying the Effects of Fish and Rhizosphere on the Structure of the Planktonic Bacterial Communities and Resistome in an Aquaponics Recirculation Loop
Source: Environ Microbiol Rep. 2025 Jul 14;17(4):e70128. doi: 10.1111/1758-2229.70128 (PMC12256928; doi:10.1111/1758-2229.70128)
Supplement: Supplementary file 1 — Figure S1. Total bacterial biomass (cells/mL) quantified by flow cytometry after Sybr Green staining within the circulating water of the different compartments of the 4 aquaponics units (n = 2–4 replicates). Figure S2. Venn diagrams showing the unique and shared ASVs of the bacterial communities between different aquaponics units. Figure S3. Rarefaction curve analysis showing the depth of 16S rRNA gene sequencing. Figure S4. Microbiome composition and relative abundances of bacterial family among sampling point and aquaponic units. Colours are scaled from highest (red) to lowest (blue) values within columns. [file EMI4-17-e70128-s001.docx]

**Supporting Information**

**Identifying the effects of fish and rhizosphere on the structure of the planktonic microbial communities and resistome in an aquaponics recirculation loop**

Frédérique Changey^a*^, Christophe Merlin^a^, Camille Fourrier^b^, Pascal Fontaine^b^, Laurence Mathieu^c^

^a^ : Université de Lorraine, CNRS, LCPME, F-54000, Nancy, France

^b :^ University of Lorraine, INRAE, L2A, 54000, Nancy, France

^c :^ EPHE, PSL, UMR CNRS 7564, LCPME, F-54000 Nancy, France

***Correspondance**

Frédérique Changey: Université de Lorraine, CNRS, LCPME, F-54000, Nancy, France ; Email :frederique.changey@univ-lorraine.fr [frederique.changey@univ-lorraine.fr](mailto:frederique.changey@univ-lorraine.fr)

# **Funding information**

# V2A project, which received financial support from the Region Grand-Est, Grant/award Number:20P11440. The experimental site was supported by EARL “La ferme de l’abbaye” (Siret: 43482106200016).


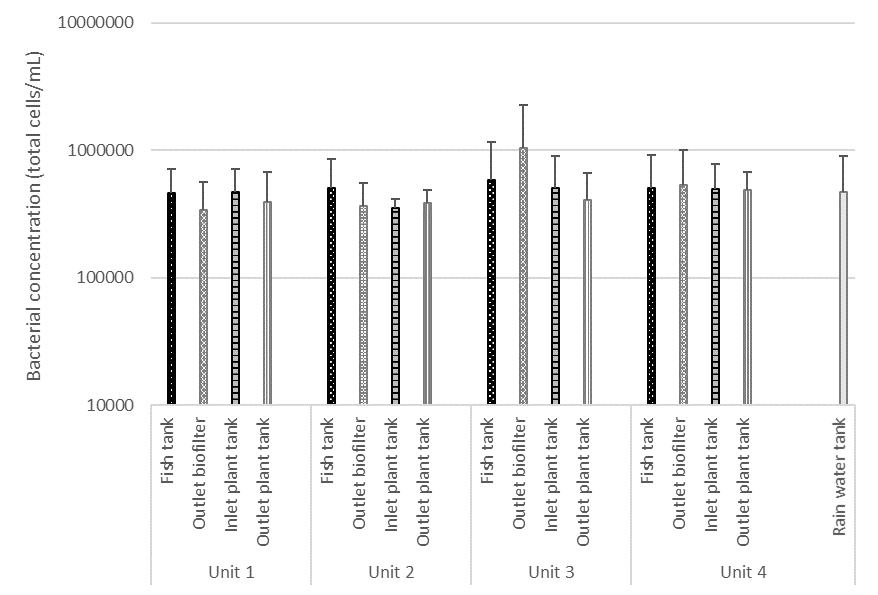


**Fig. S1** Total bacterial biomass (cells/mL) quantified by flow cytometry after Sybr-Green staining within the circulating water of the different compartments of the 4 aquaponics units. (n = 2 to 4 replicates).

**Fig. S2** Venn diagrams showing the unique and shared ASVs of the bacterial communities between different aquaponics units


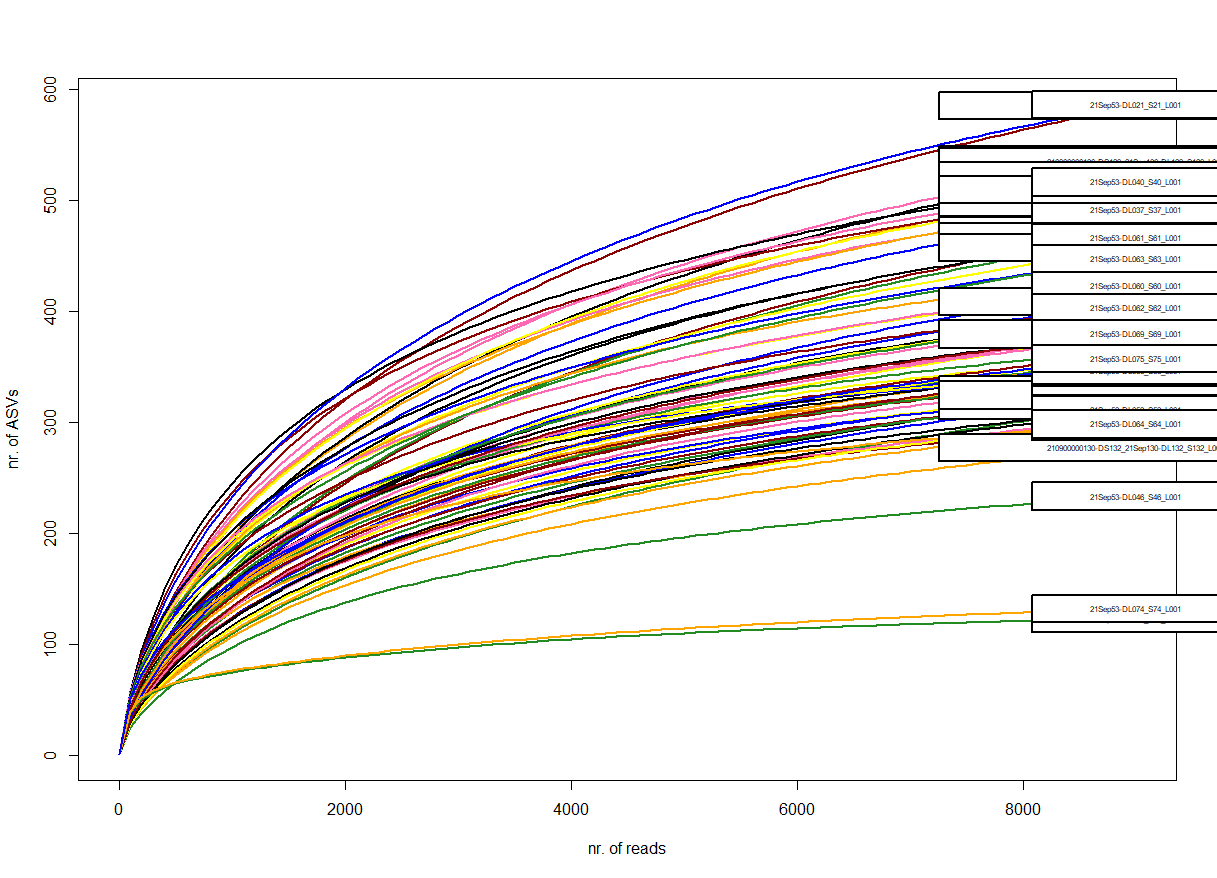


**Fig. S3** Rarefaction curve analysis showing the depth of 16S rRNA gene sequencing

**Fig. S4** Microbiome composition and relative abundances of bacterial family among sampling point and aquaponic units. Colors are scaled from highest (red) to lowest (blue) values within columns.
